# Anti-Staphylococcal Activity of *Cinnamomum zeylanicum* Essential Oil against Planktonic and Biofilm Cells Isolated from Canine Otological Infections

**DOI:** 10.3390/antibiotics11010004

**Published:** 2021-12-22

**Authors:** Vinicius de Queiroz Albuquerque, Maria Janeila Carvalho Soares, Maria Nágila Carneiro Matos, Rafaela Mesquita Bastos Cavalcante, Jesús Alberto Pérez Guerrero, Tigressa Helena Soares Rodrigues, Geovany Amorim Gomes, Rodrigo Fonseca de Medeiros Guedes, Débora de Souza Collares Maia Castelo-Branco, Isaac Neto Goes da Silva, Victor Alves Carneiro

**Affiliations:** 1 Department of Veterinary Sciences, State University of Ceará—Itaperi Campus, Fortaleza 60714-903, Brazil; viniciusqalbuquerque@gmail.com (V.d.Q.A.); isaac.neto@uece.br (I.N.G.d.S.); 2 Center for Bioprospecting and Applied Molecular Experimentation (NUBEM), University Center INTA-UNINTA, Sobral 62050-100, Brazil; janeilacarvalho29@gmail.com; 3 Laboratory of Biofilms and Antimicrobial Agents (LaBAM), Federal University of Ceará, Sobral 62048-280, Brazil; nagilacarneirobio@gmail.com (M.N.C.M.); rafaelabastos.ufc@gmail.com (R.M.B.C.); jesus24p@gmail.com (J.A.P.G.); 4 Center of Exact Science and Technology, State University of Acaraú Valley, Sobral 62040-370, Brazil; thelenasr@yahoo.com.br (T.H.S.R.); pesquisadorgeo@yahoo.com.br (G.A.G.); 5 Group of Applied Medical Microbiology, Microbiology Department, Federal University of Ceará, Fortaleza 60430-160, Brazil; fmg_rodrigo@hotmail.com (R.F.d.M.G.); deb_castelobranco@yahoo.com (D.d.S.C.M.C.-B.)

**Keywords:** natural product, antibiofilm activity, *Staphylococcus* spp., canine-ear infections

## Abstract

The aim of this study was to evaluate the phytochemical profile of *Cinnamomum zeylanicum* essential oil (CZEO) and their antimicrobial and antibiofilm activity against *Staphylococcus* strains isolated from canine otitis. First, the CZEO chemical composition was determined by gas chromatography-mass spectrometry (CG-MS). External otitis samples collected from dogs were submitted to staphylococcal isolation, followed by MALDI-TOF mass spectrometry identification. The antimicrobial action was tested against the isolates using the disk-diffusion and microdilution methods. The antibiofilm activity was evaluated by CZEO-based concentrations, subMIC for biofilm formation and supraMIC against preformed biofilm, quantified by crystal violet (CV) staining and CFU counting. The chemical analysis revealed that (E)-cinnamaldehyde, eugenol and (E)-cinnamyl acetate were the main compounds in the CZEO, representing 77.42, 8.17 and 4.50%, respectively. Two strains of three different species, *S. saprophyticus*, *S. schleiferi* and *S. pseudintermedius*, were identified. The disk-diffusion test showed an inhibitory zone diameter, ranging from 34.0 to 49.5 mm, while the MIC and MBC values were around 500 and 1000 µg/mL. SubMIC demonstrated an inhibition on biofilm formation against 4 out the 6 strains tested. On mature biofilm, the CZEO-based supraMIC groups had slightly change on biomass, however, the biofilm cell viability decreased the CFU in 3 magnitude orders.

## 1. Introduction

Otitis-related problems are one the most common diseases observed in canine patients, which cause inflammation of the ear membranes due to the uncontrolled growth of pathogenic bacteria and fungi [1]. Among bacterial agents, *Staphylococcus* strains are frequently associated with these infections, even though they are commensally present in small numbers on the skin and mucous membranes in healthy dogs [2]. When these bacteria become numerous, infections such as pyoderma and otitis media or externa may develop and prolong inflammation [3,4].

In those cases, antibiotics are the most common medication for domestic animals, yet they are also the most frequently misused. The indiscriminate use of antibiotics to prevent, control, and treat infections caused by microorganisms has allowed the emergence and spread of microbial mechanisms of resistance [5]. For instance, the prevalence of penicillin-derived antibiotics resistance in *Staphylococcus* isolates obtained from canine and feline infections, such as methicillin-resistant *Staphylococcus* (MRS), has increased continuously over the last several decades [6]. Another factor that contributes to antibiotic resistance of staphylococcal strains is the formation of biofilms [7]. These biofilms are associated with the adhesion and colonization of different surfaces that also serve as physical and chemical barriers against the action of antimicrobial agents [8]. As antibiotic resistance continues to evolve, antibiotics of so-called last resort become even more precious. Reducing or preventing the dissemination of antibiotic resistance genes into human pathogens is currently of high international importance.

To date, it has not been found a solution solve the antibiotic resistance problem. Essential oils (EO), their main constituents, and most recently their association with antibiotics have been widely studied as promising therapeutic alternatives against different species of the *Staphylococcus* genus [9,10]. Studies exploring veterinarian applications of EOs as antimicrobials to treat microorganisms that cause skin infections in dogs are incipient, but promising [11,12]. The aim of this study was to evaluate the antimicrobial and antibiofilm activity of *Cinnamomum zeylanicum* EO (CZOE) against *Staphylococcus* sp. strains isolated from dogs with otitis infection.

## 2. Results

### 2.1. Staphylococcal Strains

Two strains of three *Staphylococcal* species were isolated and identified as *S. pseudintermedius, S. schleiferi,* and *S. saprophyticus.* Five of the six isolates presented resistance for at least one antibiotic, except for *S. schleiferi* 1, which was sensitive against all the antibiotics tested. The most striking result was the strain *S. pseudintermedius* 1 which exhibited multi-resistance to five antibiotics. The *Staphylococcal* strains identification and resistance profile are described below (Table 1).

### 2.2. CZEO Chemical Composition

**Figure 1:**
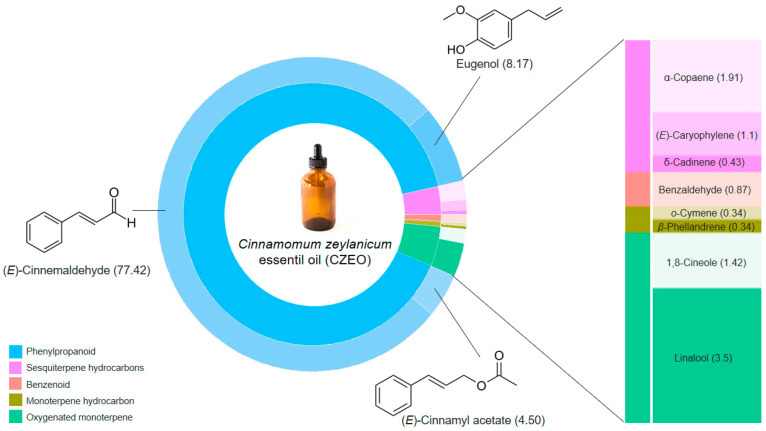
Percentage of the chemical compositions of the *Cinnamomum zeylanicum* essential oils (CZEO) and chemical structures of the main constituents.

It was identified 11 compounds, 100% of the constituents, in the CZEO. The composition and chemical structures of the main constituents are presented in Figure 1. The CZEO consisted mainly of (*E*)-cinnamaldehyde (77.42%), which together with eugenol and (*E*)-cinnamyl acetate accounted for more than 90% of the oil’s composition. The remaining fraction was constituted by oxygenated monoterpenes, sesquiterpenes hydrocarbons and traces of benzenoids and monoterpenes. The Kovats indices from the literature (KIL) and the Kovats indices calculated (KIC) against n-alkanes (C7–C30) on an RTX-5MS column are available as Appendix A.

### 2.3. Antimicrobial Activity

The CZEO demonstrated antimicrobial activity against all the *Staphylococcus* strains. The inhibitory parameters are shown in Table 2. Both *S. saprophyticus* strains showed the higher IZD, MIC, and MBC values, while *S. schleiferi* and *S. pseudintermedius* strains displayed moderate action with lower values for the same parameters.

### 2.4. Antibiofilm Activity

**Figure 2:**
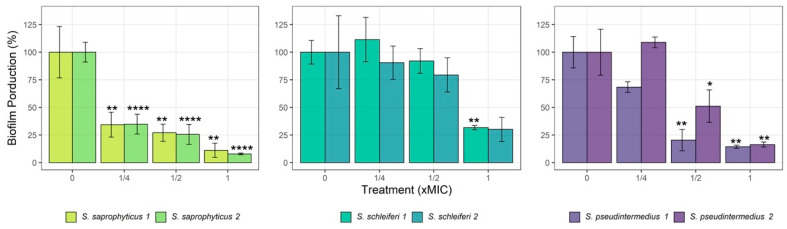
Percentage of inhibition of *Staphylococcus* biofilm formation growth simultaneously with sub-inhibitory concentration (¼-, ½- and 1-fold of MIC) of *Cinnamomum zeylanicum* essential oil (CZEO) during 24 h. Statistically different by one-way ANOVA with Tukey post hoc test (* *p* < 0.05, ** *p* < 0.01; **** *p* < 0.0001) compared to untreated cells.

On average, CZEO-treated strains at subMIC had their biofilm formation inhibited (Figure 2). Both *S. saprophyticus* strains showed the highest biomass reduction, up to 85% at 1-MIC value. Although *S. pseudintermedius* strains were less affected by the CZEO, they also showed a reduction in biomass (80 and 50%) when treated with EO at ½ and 1-MIC values. Finally, the results did not reveal significance difference for *S. schleiferi* strains at subMIC concentrations.

**Figure 3:**
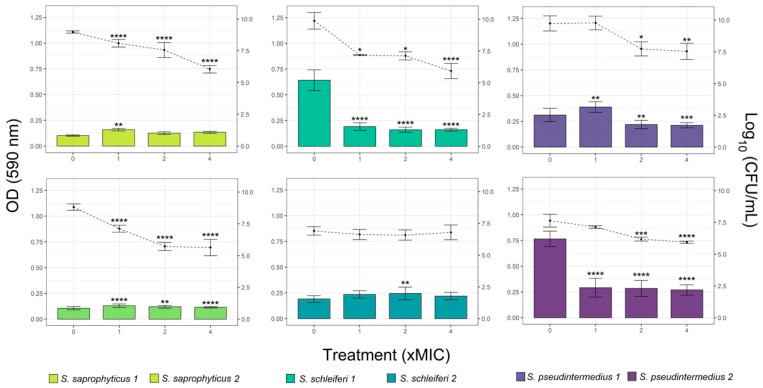
Activity of different concentrations (1-, 2- and 4-fold of MIC) of *Cinnamomum zeylanicum* essential oil (CZEO) against *Staphylococcus* preformed biofilm after 4 h of treatment. Biomass quantification by crystal violet staining (OD 590 nm, bars) and colony counting (log_10_ CFU/mL, lines). Statistically different by one-way ANOVA with Tukey post hoc test (* *p* < 0.05; ** *p* < 0.01; *** *p* < 0.001; **** *p* < 0.0001) compared to untreated cells.

According to preformed biofilm, overall, there was a positive correlation between the CZEO concentration and the reduction of biomass and viable cells of biofilm (Figure 3). The results showed that 4 of the 6 strains reduced their biomass when treated with CZEO. Interestingly, high concentration of CZEO did not decrease the biomass of *S. saprophyticus* 1 and *S. schleiferi* 2. Substantial reduction of viable cells, up to three orders in magnitude, was found for both *S. saprophyticus* and *S. schleiferi* 1 at 4-fold MIC. While, similar results were reported for both *S. pseudintermedius* strains, no signs of reduction in CFU counting was found for *schleiferi* 2.

## 3. Discussion

This study shows that CZEO was composed mostly by phenylpropanoids, specifically (*E*)-cinnamaldehyde (77.42%), eugenol (8.17%), and (*E*)-cinnamyl acetate (4.50%). These results agreed with the major components reported for CZEO chemical composition [11,12]. The potential use of (*E*)-cinnamaldehyde and eugenol, regardless their origin, has been quite explored due to significant broad-spectrum activity against pathogenic bacteria [13,14]. Their action is frequently associated with the modulation of the structural properties of the cell surface, permeability and damaged, whether cell wall or plasmatic membrane level [15,16,17], although the contribution of the non-major components in the essential oil’s antimicrobial effect still remains unclear.

The indiscriminate use of antibiotics in both human and veterinary medicine has caused a chain reaction for the appearance of multidrug-resistant (MDR) microorganisms to conventional treatments [18]. Not only all staphylococcal species isolated in this study are frequently described as etiological agents of ear canine infection [19], but also five out of the six strains were resistant to at least one antibiotic and three of them displayed a multidrug-resistance profile. The CZEO antimicrobial activity ranged from 500 to 1000 µg/mL and is consistent with previous results from the *Cinnamomum* genus, which can reach until 1250 µg/mL [20]. Although the antimicrobial effect can be associated with the presence of the major components of EO, or even an interaction between their components; further work needs to be done to establish exactly the mechanism of action and the components responsible for the antimicrobial effect.

Despite the efforts on exploring essential oils and their antimicrobial activity, the antibiofilm effect has been less evaluated [10,21,22,23]. In this study, we analyzed the CZEO ability to inhibit biofilm formation and we found that CZEO interfered on most staphylococcal biofilm, five of six strains tested (Figure 2). This action can be related to the antimicrobial action against planktonic cells in the first inoculum, resulting in a lower cell viability and minor biofilm attachment [24,25]. Nevertheless, should be considered the cinnamaldehyde effect on quorum sensing, system of bacterial communication that regulates the biofilm formation, previously reported in *S. aureus* strains [26]. Thus, this molecule could also play an important anti-biofilm activity, since it reaches more than 77 % of CZEO composition.

The physical tolerance promoted mainly by EPS (exopolysaccharide) production is crucial to restrict the penetration and diffusion of antimicrobials, protecting the cells against hostile environment [8]. Thus, the antimicrobial ability against preformed (mature) biofilm is an important parameter to measure promising approaches for biofilm eradication [27]. Regarding to mature biofilm, our results showed that four of the six strains reduced their biomass when treated with CZEO. Interestingly, the strains with the highest biofilm production, *S. schleiferi* 1 and *S. saprophyticus* 2, displayed more reductions in biomass with values above 60%, even when treated with lower EO concentration (Figure 3). Although there are several possible explanations for this outcome, we suggest that larger tridimensional biofilm structures may be more impacted by antibiofilm agents due to increase in surface area increasing the direct killing of biofilm-embedded bacteria, as it has been demonstrated in other studies [16,28].

In addition to the mature biofilm reduction, the CFU counting shows that EO effect is directly related to bacterial death, being proportional to the oil concentration (Figure 3). This is relevant since some cells into biofilm deep layers can show a persistence profile, also known as slow-dividing bacteria, and consequently are less susceptible to antibiotics [29]. Thus, the findings support the idea that natural-based compounds are a great source of new strategies to fight against resistant bacteria and eliminate microbial biofilms [30]. Studies relating natural plant products as new alternatives to combat MDR bacteria have gained researchers attention to develop creams and gels for topical applications [31,32]. EOs, widely commercialized, have become a viable alternative for the development of new treatments against skin infections in veterinary medicine, including pyoderma, media- and external-otitis [11].

## 4. Materials and Methods

### 4.1. Staphylococcal Strains

Ear-secretions samples from dogs with clinical signs for otitis externa were collected using sterile cotton swabs in a veterinary clinic localized in Fortaleza, Brazil. The samples were initially inoculated on blood agar and incubated aerobically for 24 h at 37 °C. Suspected colonies were submitted to presumptive *Staphylococcus* identification through selective medium culture, Mannitol Salt Agar (KASVI, São José dos Pinhais, Paraná, Brazil) and Baird Parker Agar (Acumedia, Lansing, MI, USA), colony morphology, Gram staining and coagulase test. The isolates were finally submitted to a Matrix-Assisted Laser Desorption/Ionization—MALDI (Bruker Daltonik MALDI Biotyper) mass spectrometry identification [33]. The isolates resistance profile was determined by the disk diffusion method against antibiotics panel, following the protocols of the Clinical and Laboratory Standards Institute [34]. The strains were stored in Tryptic Soy Broth (TSB, KASVI, São José dos Pinhais, Paraná, Brazil) with glycerol at −20 °C.

### 4.2. Culture Conditions

From stock cultures, the strains were activated inoculating 50 μL into 5 mL of TBS and incubated at 37 °C for 24 h in aerobic conditions. Next, 50 μL were inoculated in fresh medium (1:100) and grown until the end of the exponential phase. Bacterial cells were adjusted compared to the McFarland 0.5 turbidity standard (1 × 10^8^ CFU/mL) and diluted with suitable media to reach the appropriate cell concentration for each method.

### 4.3. Oil Acquisition and Manipulation

The CZOE was purchased from Laszlo^®^ aromatherapy company (Belo Horizonte, Brazil). According to the manufacturer, the CZEO was extracted from the stem bark of *C. zeylanicum* plant from Sri Lanka using steam distillation. CZEO stock solution was prepared at 16,000 µg/mL in sterile TSB at 1% tween 80 (Sigma Aldrich^®^, Saint Louis, MO, USA). Free-EO TSB at 1% tween 80 was used as control.

### 4.4. Determination of CZEO Chemical Composition

The volatile oil was analyzed by gas chromatography-mass spectrometry (GC-MS) and gas chromatography with flame ionization detection (GC-FID). The qualitative and quantitative analysis of the oil was performed using a Shimadzu single quadrupole GCMS-QP2010 gas chromatograph coupled to VG Analytical 70–250 S mass spectrometer and a flame ionization detector (FID). The percentage of constituents was calculated through the integral area of their respective peaks, related to the total area of all constituents in the sample. The different EOs’ constituents were identified by visually comparing their mass spectra provided by the equipment’s databases (NIST08) as well as by comparison of the calculated retention indices to those available in the literature [35]. A standard solution of n-alkanes (C7–C30) was injected under the same chromatographic conditions as the sample and was used to obtain the retention indices as described by Van den Dool and Kratz [36].

### 4.5. CZEO Antimicrobial Activity

#### 4.5.1. The Aromatogram Method

The CZEO antimicrobial activity was performed as in traditional antibiotic susceptibility testing using the disc diffusion test [37], known as the aromatogram method [38]. Cotton-tipped swabs dipped into a standardized bacterial suspension adjusted at 1 × 10^8^ CFU/mL were streaked on Mueller Hinton agar plates (MHA, Acumedia^®^, Lansing, MI, USA) to cover the entire surface. After plate drying, 6 mm diameter sterile paper disks, previously impregnated with 10 µL of pure CZEO, were placed on the plates and incubated for 24 h at 37 °C. The diameter of the inhibition zone was measured in millimeters using a caliper.

#### 4.5.2. Minimum Inhibitory Concentration (MIC) and Minimum Bactericidal Concentration (MBC)

MICs values were determined using the broth microdilution assay in 96-well polystyrene microtiter plates with round bottom (KASVI, São José dos Pinhais, Paraná, Brazil). First, all wells were filled with 100 μL of CZEO prepared in TSB in a 2-fold reduction concentration from 4000 to 125 µg/mL. Then, 100 µL of the bacterial suspension at 1 × 10^6^ CFU/mL were added to each well, and the plate was incubated at 37 °C under aerobic conditions. After 24 h, the lowest concentration of EO that completely inhibited the bacterial growth was considered as MIC [36]. MBCs were determined in broth dilution tests by sub-culturing 10 μL from the wells where there was no visible microbial growth to plates containing TSB Agar. The plates were incubated for 24 h at 37 °C under aerobic conditions. Then, the lowest concentration of CZEO that decreased 99.9% of the bacterial population was considered as MBC.

### 4.6. CZEO Antibiofilm Activity

#### 4.6.1. Biofilm Formation

The CZEO activity to inhibit the biofilm formation was analyzed as the minimal concentration able to prevent the initial adhesion and biofilm development on polystyrene surface (O’Toole, 2011). A serial 2-fold dilutions of CZEO at sub inhibitory concentrations (subMIC, ¼-, ½- and 1-fold MIC) were prepared at 100 μL of final volume in 96 well flat-bottom polystyrene microtiter plates. Next, 100 μL of a cell suspension at 10^6^ CFU/mL prepared in the TSB was added into each well and incubated at 37 °C for 24 h under aerobic conditions.

The biofilm biomass was quantified by crystal violet (CV) staining. Firstly, each well was washed thrice with sterile distilled water to remove planktonic cells. After aspiration of unattached cells, biofilms were fixed with 200 µL of 95% methanol (Dinâmica, São Paulo, Brazil) for 10 min and air-dried at room temperature. Then, 200 μL of the 0.1% CV solution (Synth^®^, São Paulo, Brazil) were added and incubated for 10 min. The plate was washed three times with saline, and the biomass-linked dye was solubilized in 200 μL of 33 % glacial acetic acid (Dinâmica, São Paulo, Brazil). The biofilm was indirectly quantified by measuring the supernatant absorbance after CV solubilization at 590 nm using an optical density reader (SpectraMax^®^ Paradigm^®^ Molecular Devices, San Jose, CA, USA) at 590 nm.

#### 4.6.2. Preformed Biofilm

The mature *Staphylococcus* biofilm susceptibility to CZEO was evaluated in 96-well polystyrene flat-bottomed microtiter plates. After biofilm formation under aerobic conditions during 24 h with the 200 µL of initial inoculum of 1 × 10^6^ CFU/mL, the wells were washed three times with saline solution to remove the planktonic and weakly attached cells. Subsequently, the biofilm was treated with 200 µL CZEO solutions, ranging from subMIC to 1, 2 and 4-fold MIC. After 24 h at 37 °C, the content of each well was removed, washed with saline, and the residual biofilm was quantified according to biomass and the number of viable cells by CV staining (described before) and colony forming units (CFU), respectively.

To determine the viable cells in the CZEO-treated biofilm, 200 µL of saline solution were added to each well, and the microplate was subjected to ultrasound (GNATUS, São Paulo, Brazil) for 5 min to detach the biofilm-embedded bacteria. A serial ten-fold dilutions were prepared from the cell suspension, and 10 μL were plated in TSB agar. After 24 h of incubation at 37 °C, the total of enumerated cells was expressed in log_10_ CFU/mL from the average of the number of CFUs of three different wells from the same replicate.

### 4.7. Statistical Analyses

All microbial assays were performed in triplicate on three different days, and the results were presented as the mean± standard deviations (SD). Statistical analysis was performed by a one-way analysis of variance (ANOVA) and Tukey post-hoc test using free software R Studio (Integrated Development for R. PBC, Boston, MA, USA). Differences between groups compared to untreated cells were considered significant for * *p* < 0.05, ** *p* < 0.01, *** *p* < 0.001, **** *p* < 0.0001.

## 5. Conclusions

We have found that CZEO has an effective antimicrobial and antibiofilm activity against Staphylococcal strains and could represent a potential alternative to treat common ear infections in canines caused by *Staphylococcus* spp. However, more studies are needed to characterization the mechanism of action as well as to standardize the best way to use it against the pathogenic bacteria.

## Figures and Tables

**Table 1:** *Staphylococcus* isolates identification and resistance profile by MALDI-TOF mass spectrometry and disk-diffusion assay, respectively.

Sample	MALDI-TOF ID	Resistance Profile
1	*S. saprophyticus* 1	PEN
2	*S. saprophyticus* 2	AZI, ERI, CLO
3	*S. schleiferi* 1	
4	*S. schleiferi* 2	SUT
5	*S. pseudintermedius* 1	CIP, SUT, TET, PEN, CLI
6	*S. pseudintermedius* 2	TET, CLO

Antibiotics panel: PEN: penicillin. AZI: azithromycin. ERI: erythromycin. CLO: chloramphenicol. SUT: sulfazotrim. GEN: gentamicin. CIP: ciprofloxacin. TET: tetracycline. CLI: clindamycin.

**Table 2:** Inhibitory zone diameter (IZD), minimum inhibitory concentration (MIC) and minimum bactericidal concentration (MBC) of the *Cinnamomum zeylanicum* essential oil (CZEO) for the *Staphylococcus* strains.

*Staphylococcus* Strains	IZD (mm)	MIC (µg/mL)	MBC (µg/mL)
*S. saprophyticus* 1	49.5 ± 2.1	1000	1000
*S. saprophyticus* 2	49.0 ± 0.1	1000	1000
*S. schleiferi* 1	34.0 ± 2.0	500	500
*S. schleiferi* 2	39.0 ± 2.8	500	500
*S. pseudintermedius* 1	35.0 ± 4.2	500	500
*S. pseudintermedius* 2	36.0 ± 0.1	500	500

## Appendix A

**Table A1:** Percentage of the *Cinnamomum zeylanicum* Essential Oil Chemical Compositions.

**Identified Components**	**KIC ^1^**	**KIL ^2^**	**CZEO (%) ^3^**
*Benzenoid*			*0.87*
Benzaldehyde	967	960	0.87
*Monoterpene hydrocarbons*			*0.68*
o-cimene	1030	1026	0.34
β- Phellandrene	1036	1029	0.34
*Oxygenated monoterpene*			*4.92*
1,8-Cineol	1039	1031	1.42
linalool	1102	1096	3.5
*Phenylpropanoids*			*90.09*
(*E*)-cinnamaldehyde	1281	1270	77.42
eugenol	1364	1359	8.17
(*E*) cinamyl-acetato	1451	1446	4.5
*Sesquiterpene hydrocarbons*			*3.44*
α- Copaene	1383	1376	1.91
(*E*)-Caryophylene	1428	1419	1.1
δ-Cadinene	1531	1523	0.43
**Total identified**			100

^1^ KIC: Kovats indices calculated against n-alkanes (C7–C30) on an RTX-5MS column. ^2^ KIL: Kovats retention indexes in the literature (ADAMS, 2007). ^3^ Percentage of the peak area in relation to the total area of the sample.
